# High Serum Caspase-Cleaved Cytokeratin-18 Levels and Mortality of Traumatic Brain Injury Patients

**DOI:** 10.3390/brainsci9100269

**Published:** 2019-10-10

**Authors:** Leonardo Lorente, María M. Martín, Agustín F. González-Rivero, Antonia Pérez-Cejas, Mónica Argueso, Luis Ramos, Jordi Solé-Violán, Juan J. Cáceres, Alejandro Jiménez, Victor García-Marín

**Affiliations:** 1 Intensive Care Unit, Hospital Universitario de Canarias, Ofra, s/n. La Laguna, 38320 Santa Cruz de Tenerife, Spain; 2 Intensive Care Unit, Hospital Universitario Nuestra Señora de Candelaria, Crta del Rosario s/n, 38010 Santa Cruz de Tenerife, Spain; mar.martinvelasco@gmail.com; 3 Laboratory Department, Hospital Universitario de Canarias, Ofra, s/n. La Laguna, 38320 Santa Cruz de Tenerife, Spain; agonriv@hotmail.com (A.F.G.-R.); aperezcejas@gmail.com (A.P.-C.); 4 Intensive Care Unit, Hospital Clínico Universitario de Valencia, Avda. Blasco Ibáñez n 17–19, 46004 Valencia, Spain; moni_begasa@hotmail.com; 5 Intensive Care Unit, Hospital General de La Palma, Buenavista de Arriba s/n, Breña Alta, 38713 La Palma, Spain; lramosgomez@gmail.com; 6 Intensive Care Unit, Hospital Universitario Dr. Negrín, CIBERES, Barranco de la Ballena s/n, 35010 Las Palmas de Gran Canaria, Spain; jsolvio@gobiernodecanarias.org; 7 Intensive Care Unit, Hospital Insular, Plaza Dr. Pasteur s/n, 35016 Las Palmas de Gran Canaria, Spain; juanjose.caceresagra@gobiernodecanarias.org; 8 Research Unit, Hospital Universitario de Canarias, Ofra, s/n, La Laguna, 38320 Santa Cruz de Tenerife, Spain; ajimenezsosa@gmail.com; 9 Department of Neurosurgery, Hospital Universitario de Canarias, Ofra, s/n. La Laguna, 38320 Santa Cruz de Tenerife, Spain; vicgarmar666@gmail.com

**Keywords:** cytokeratin, brain trauma, patients, mortality, injury

## Abstract

Objective: Apoptosis increases in traumatic brain injury (TBI). Caspase-cleaved cytokeratin (CCCK)-18 in blood during apoptosis could appear. At the time of admission due to TBI, higher blood CCCK-18 levels were found in non-surviving than in surviving patients. Therefore, the objective of our study was to analyze whether serum CCCK-18 levels determined during the first week after TBI could predict early mortality (at 30 days). Methods: Severe TBI patients were included (considering severe when Glasgow Coma Scale < 9) in this observational and multicentre study. Serum CCCK-18 levels were determined at day 1 of TBI, and at days 4 and 8 after TBI. Results: Serum CCCK-18 levels at day 1 of TBI, and in the days 4 and 8 after TBI were higher (*p* < 0.001) in non-surviving than in surviving patients (34 and 90 patients, respectively) and could predict early mortality (*p* < 0.001 in the area under the curve). Conclusions: The new findings from our study were that serum CCCK-18 levels at any moment of the first week of TBI were higher in non-surviving patients and were able to predict early mortality.

## 1. Introduction

Many disabilities and deaths are due to traumatic brain injury (TBI) [1]. A secondary brain injury could appear within hours or days after a TBI due to apoptosis [2,3,4,5]. The programmed cell death by apoptosis is increased in TBI according to the findings in brain samples of animals [6,7,8] and humans [9,10].

The cytokeratin protein group is present mainly in the intracytoplasmic cytoskeleton filaments of epithelial tissue and participates in static cell functions (such as tensile strength) and dynamic cell processes (such as mitosis, differentiation, and movement) [11]. Caspase-cleaved cytokeratin (CCCK)-18 appears in the bloodstream due to the action of caspases on cytokeratin-18 during apoptosis [12]. A worse prognosis has been found in patients with different diseases such as sepsis [13] or hepatocellular carcinoma [14], and high circulating levels of CCCK-18.

Regarding CCCK-18 and cerebral processes, CCCK-18 levels have been found in brain samples of rats with glioma [15] and in patients with pituitary adenomas [16]. In addition, higher circulating levels of CCCK-18 have been found in patients with worse prognosis after cerebral hemorrhage [17,18,19] or cerebral infarction [20]. Furthermore, higher blood levels of CCCK-18 have been found at the time of admission of TBI in 30-day non-surviving than in surviving patients [21]. Therefore, the objective of our study was to analyze whether serum CCCK-18 levels determined during the first week after TBI could predict early mortality (at 30 days).

## 2. Methods

### 2.1. Design and Subjects

Six intensive care units from Spanish hospitals participated in this prospective and observational study. The Institutional Review Board of all hospitals approved the protocol study: H. Universitario Dr. Negrín of Las Palmas de Gran Canaria, H. Universitario Nuestra Señora de Candelaria of Santa Cruz de Tenerife, H. Insular de Las Palmas de Gran Canaria, H. General de La Palma, H. Clínico Universitario de Valencia, and H. Universitario de Canarias of La Laguna. The legal guardians of each patient signed the written informed consent for the participation in the study.

Only patients with severe TBI, defined as <9 points in the Glasgow Coma Scale (GCS) [22], and with only <10 points in non-cranial aspects of the Injury Severity Score (ISS) [23] were included. Pregnant patients, patients under 18 years of age, and patients with inflammatory disease or malignant disease or comfort measures only were excluded.

We had previously measured serum CCCK-18 levels at the time of admission for TBI or day 1 of TBI (within 4 hours of the TBI) in some of these patients [21], and in our current research, serum CCCK-18 levels were measured on day 1 of the TBI, and on days 4 and 8 after the TBI.

The following were collected from each patient: glycemia, bilirubin, lactic acid, pressure of arterial oxygen (PaO_2_)/fraction inspired oxygen (FIO_2_) ratio, creatinine, sodium, hemoglobin, platelets, leukocytes, fibrinogen, activated partial thromboplastin time (aPTT), and international normalized ratio (INR). In addition, ISS, sex, age, cerebral perfusion pressure (CPP), intracranial pressure (ICP), GCS, Acute Physiology and Chronic Health Evaluation II (APACHE II) score [24], and classification of Marshall computer tomography for head injury (CT) [25] were recorded. Thirty-day mortality was the end-point of the study.

### 2.2. Serum CCCK-18 Analysis

Serum samples were obtained on day 1 of TBI, and on days 4 and 8 after TBI, and were frozen at –80 °C until serum determinations. CCCK-18 concentrations were determined using the kit M30 Apoptosense® (PEVIVA AB, Bromma, Sweden). The inter-assay coefficient of variation, detection limit, and intra-assay coefficient of variation were <10%, 25 µ/L, and <10%, respectively.

### 2.3. Statistical Methods

Continuous variables such as medians (and interquartile ranges) were recorded. We used the Kolmogorov–Smirnov test to compare empirical distributions with the normal distribution. As serum CCCK-18 levels were not adjusted to normal distribution, we then compared them using intergroups of patients (between survivors and non-survivors) by Wilcoxon–Mann–Whitney tests and intragroups of patients (in survivors and in non-survivors) by paired sample Wilcoxon tests. Categorical variables such as frequencies (and percentages) were recorded and compared between groups of patients using the chi-square test. Receiver operating characteristic (ROC) analyses with area under curve (AUC) were performed and specificity, sensitivity, negative predicted value, negative likelihood ratio, positive predicted value, and positive likelihood ratio of optimal cut-offs of serum CCCK-18 concentrations (selected according to Youden J index) were reported on day 1 of TBI, and on days 4 and 8 after TBI for 30-day mortality prediction with 95% confidence intervals (CI). A multiple logistic regression analysis was performed to determine the association between serum CCCK-18 levels and 30 day-mortality, controlling for CT, sex, and APACHE-II score. Spearman’s rho correlation coefficient was used ti test the association between continuous variables. Bonferroni correction for multiple comparisons was used. The programs NCSS 2000 (Kaysville, UT, USA), LogXact 4.1 (Cytel Co., Cambridge, MA, USA), and SPSS 17.0 (SPSS Inc., Chicago, IL, USA) were used to carry out the statistical analyses, and only *p*-values < 0.05 were considered statistically significant.

## 3. Results

Non-survivor (*n* = 34) and survivor (*n* = 90) patients did not differ with regard to glycemia, bilirubin, lactic acid, PaO_2_, PaO_2_/FIO_2_ ratio, sodium, creatinine, hemoglobin, platelets, leukocytes, fibrinogen, INR, aPTT, ISS, ICP, and CPP. Differences were found between surviving and non-surviving patients in brain computer tomography findings. Surviving patients compared to non-surviving patients showed a lower female rate, higher GCS, lower APACHE-II score, and younger age (Table 1).

**Figure 1:**
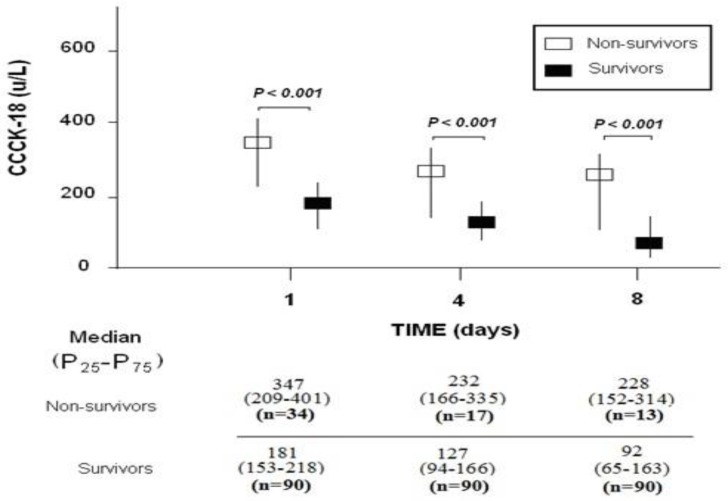
Serum levels of caspase-cleaved cytokeratin (CCCK)-18 on day 1, day 4, and day 8 after trauma brain injury in survivor and non-survivor patients.

Serum CCCK-18 levels on day 1 of TBI (*p* < 0.001), and on days 4 (*p* < 0.001) and 8 (*p* < 0.001) after TBI were higher in non-surviving than in surviving patients (Figure 1). Serum CCCK-18 levels were statistically higher on day 1 than on day 4 (*p* < 0.001) and on day 1 than on day 8 (*p* < 0.001) in surviving patients. Serum CCCK-18 levels were not statistically different between day 1 and 4 (*p* = 0.06) and between day 1 and 8 (*p* = 0.51) in non-surviving patients. After Bonferroni correction for multiple comparisons, only *p*-values < 0.007 (0.05/7) were considered statistically significant.

**Figure 2:**
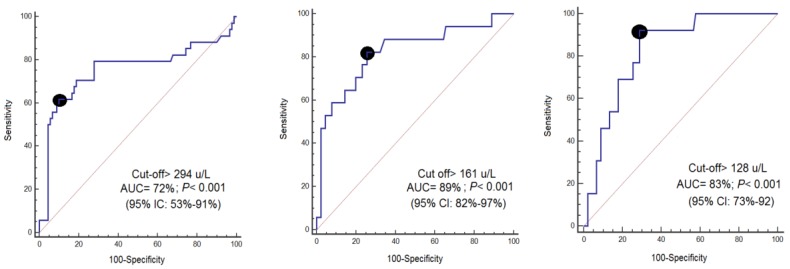
Receiver operation characteristic analysis using serum caspase-cleaved cytokeratin (CCCK)-18 levels on day 1, day 4, and day 8 after trauma brain injury as predictor of mortality at 30 days.

The AUC (and 95% CI) of serum CCCK-18 concentrations on day 1 of TBI, and on days 4 and 8 after TBI for 30-day mortality prediction were 0.75 (0.67–0.83; *p* < 0.001), 0.82 (0.73–0.89; *p* < 0.001), and 0.83 (0.74–0.90; *p* < 0.001) (Figure 2). Table 2 shows specificity, sensitivity, negative predicted value, negative likelihood ratio, positive predicted value, and positive likelihood ratio of cut-offs of serum CCCK-18 concentrations on day 1 of TBI, and on days 4 and 8 after TBI for 30-day mortality prediction.

We did not find significant differences in serum CCCK-18 concentrations according to CT (*p* = 0.78) and sex (*p* = 0.27), and neither did we find an association between serum CCCK-18 concentrations and age (rho = −0.05; *p* = 0.61). We found an association of serum CCCK-18 concentrations with GCS (rho = −0.28; *p* = 0.001) and with APACHE-II score (rho = 0.18; *p* = 0.06). Therefore, we included sex, CT, and APACHE-II score in multiple logistic regression analysis.

Multiple logistic regression analysis found an association between serum CCCK-18 concentrations and mortality (OR = 1.02; 95% CI = 1.01–1.03; *p* < 0.001) controlling for sex, CT, and APACHE-II score (Table 3). CT findings were included in the regression analysis as CT with low risk of death (types II and V) and with high risk of death (types III, IV, and VI). This classification was used because the following mortality rates were found according to the type of CT brain injury: 16.7% (5/30) in type II, 28.6% (6/21) in type III, 40.9% (9/22) in type IV, 15.8% (6/38) in type V, and 61.5% (8/13) in type VI.

## 4. Discussion

Previously, we determined blood CCCK-18 levels on admission of TBI, and we found higher blood CCCK-18 levels on admission of severe TBI in 30 days non-surviving than in surviving patients [21]. Thus, the new aspects of our current study were that blood CCCK-18 levels were determined also at days 4 and 8 of TBI. Therefore, the novel findings of our current study were that blood CCCK-18 levels at days 4 and 8 of TBI were also higher for the 30-day non-surviving than in surviving patients. Another new finding was that blood CCCK-18 levels also on days 4 and 8 of TBI could be used as predictor biomarkers of 30-day mortality. We think it very interesting that the clinician can have a biomarker that could help predict the outcome of those patients at any moment during the first week of TBI. These findings in TBI patients are consistent with other studies on cerebral hemorrhage [17,18,19] or cerebral infarction [20] patients and a worse prognosis with high serum CCCK-18 levels.

In addition, serum CCCK-18 levels were statistically lower on day 4 than on day 1, and on day 8 than on day 1 in surviving patients, and there were no statistical differences in non-surviving patients. We think that those findings may be due to a decrease in the apoptosis degree overall during the days following TBI in non-surviving patients. However, apoptosis degree is persistently higher in non-surviving patients compared to surviving patients.

We found that non-surviving patients with respect to surviving patients also showed higher female rate, higher APACHE-II score, lower GCS, higher age, and different brain computer tomography findings. According to multiple logistic regression analysis, each increase of 1 µ/L in serum CCCK-18 levels was associated approximately with an increase of 2% in mortality, each increase of 1 point in APACHE-II score was associated approximately with an increase of 38% in mortality, and females showed approximately 6 times more death than males. However, the objective of our study was to analyze whether serum CCCK-18 levels determined during the first week after TBI could help in the prediction of early mortality but not replace other variables associated with mortality. In fact, we found that serum CCCK-18 concentrations were associated with mortality controlling for sex, CT, and APACHE-II score. The advantages of serum CCCK-18 level determination with respect to CT are that it is cheaper and easier.

The meaning of these high bloodstream CCCK-18 levels in non-surviving TBI patients is unclear. Cytokeratin-18 is present in the intracytoplasmic cytoskeleton of epithelial tissue [11,12], but CCCK-18 has also been found in the brain of rats with glioma [15] and in the brain of patients with pituitary adenomas [16]. Besides, high circulating levels of CCCK-18 and a worse prognosis in patients with cerebral hemorrhage [17,18,19] or cerebral infarction [20] have been found. In addition, TBI could produce a systemic inflammatory response syndrome (SIRS) [26], which could activate apoptosis by the action of different pro-inflammatory cytokines [27].

Another interesting point is that the use of apoptosis inhibitor agents in animal models has been associated with a reduction in brain apoptosis [28,29,30,31,32,33,34,35]. Therefore, we think that all those finding could generate research about apoptosis in TBI patients and the use of apoptosis inhibitor agents.

We want to recognize as limitations in our study that we have not analyzed apoptosis and concentrations of CCCK-18 in brain samples, nor concentrations of CCCK-18 in cerebrospinal fluid, and it could be interesting to explore the association between blood CCCK-18 levels and all those aspects of apoptosis. In addition, the determination of blood CCCK-18 levels during all follow-up (30 days) and not only during the first week of TBI, could also be interesting. Furthermore, we have not monitored electrocorticography to assess the presence of cortical spreading depolarizations or epileptic events; in fact, cortical spreading depolarizations has been found in TBI and has been associated with TBI outcomes [36,37] and with apoptosis [38].

## 5. Conclusions

The new findings from our study were that serum CCCK-18 levels at any moment of the first week of TBI were higher in non-surviving patients and were able to predict early mortality.

## Figures and Tables

**Table 1:** Surviving and non-surviving patient characteristics on day 1 of trauma brain injury.

	Survivors (*n* = 90)	Non-Survivors (*n* = 34)	*p*-Value
Brain CT injury—*n* (%)			0.01
Diffuse injury I	0	0	
Diffuse injury II	25 (27.8)	5 (14.7)	
Diffuse injury III	15 (16.7)	6 (17.6)	
Diffuse injury IV	13 (14.4)	9 (26.5)	
Evacuated mass lesion V	32 (35.6)	6 (17.6)	
Non-evacuated mass lesion VI	5 (5.6)	8 (23.5)	
Brain CT with high death risk (III, IV, VI)—*n* (%)	33 (36.7)	23 (67.6)	0.002
Gender female—*n* (%)	15 (16.7)	13 (38.2)	0.02
Decompressive craniectomy—*n* (%)	13 (14.4)	5 (14.7)	0.99
Age (years)—m (p 25–75)	46 (28–62)	65 (55–75)	<0.001
Sodium (mEq/L)—m (p 25–75)	140 (138–143)	141 (136–147)	0.41
Glycemia (g/dL)—m (p 25–75)	139 (121–167)	160 (125–191)	0.11
Lactic acid (mmol/L)—m (p 25–75)	1.75 (1.10–2.50)	2.30 (1.25–4.58)	0.08
Bilirubin (mg/dL)—m (p 25–75)	0.60 (0.40–0.80)	0.70 (0.53–1.05)	0.06
Creatinine (mg/dL)—m (p 25–75)	0.80 (0.70–1.00)	0.80 (0.70–1.10)	0.50
INR—m (p 25–75)	1.11 (1.00–1.24)	1.12 (1.03–1.48)	0.19
Platelets—m × 10^3^/mm^3^ (p 25–75)	182 (135–238)	172 (125–232)	0.49
aPTT (seconds)—m (p 25–75)	28 (25–31)	29 (25–37)	0.25
PaO_2_/FI0_2_ ratio—m (p 25–75)	336 (246–400)	294 (167–395)	0.11
PaO_2_ (mmHg)—m (p 25–75)	148 (110–242)	142 (97–195)	0.45
Leukocytes—m × 10^3^/mm^3^ (p 25–75)	13.9 (10.1–19.0)	14.9 (9.7–21.6)	0.47
Fibrinogen (mg/dL)—m (p 25–75)	371 (286–471)	348 (300–475)	0.70
Hemoglobin (g/dL)—m (p 25–75)	11.2 (10.0–13.0)	11.9 (10.0–13.7)	0.73
Glasgow Coma Scale—m (p 25–75)	7 (5–8)	4 (3–7)	<0.001
ICP peak (mmHg)—m (p 25–75)	15 (14–20)	25 (11–30)	0.08
CPP small (mmHg)—m (p 25–75)	68 (57–70)	61 (52–70)	0.20
APACHE-II—m (p 25–75)	18 (14–22)	25 (23–28)	<0.001
ISS—m (p 25–75)	25 (25–29)	25 (25–26)	0.59
CCCK-18 (µ/L)—m (p 25–75)	181 (153–218)	347 (209–401)	<0.001

CT = computer tomography; m = median; p 25–75 = percentile 25th–75th; INR = international normalized ratio; aPTT = activated partial thromboplastin time; PaO_2_ = pressure of arterial oxygen; FIO_2_ = fraction inspired oxygen; ICP = intracranial pressure; CPP = cerebral perfusion pressure; APACHE II = Acute Physiology and Chronic Health Evaluation; ISS = Injury Severity Score; CCCK = caspase-cleaved cytokeratin.

**Table 2:** Receiver operation characteristic analysis using serum caspase-cleaved cytokeratin (CCCK)-18 levels on day 1, day 4, and day 8 after trauma brain injury as predictor of mortality at 30 days.

	Day 1	Day 4	Day 8
Cut-off of CCCK-18 (µ/L)	>294	>161	>128
Specificity (95% CI)	90% (82–95%)	74% (64–83%)	71% (61–80%)
Sensitivity (95% CI)	62% (44–78%)	82% (57–96%)	92% (64–99%)
Negative likelihood ratio (95% CI)	0.4 (0.3–0.7)	0.2 (0.1–0.7)	0.1 (0.02–0.70)
Positive likelihood ratio (95% CI)	6.2 (3.1–12.1)	3.2 (2.1–4.9)	3.3 (2.2–4.6)
Negative predicted value (95% CI)	86% (80–91%)	96% (89–98%)	98% (91–99%)
Positive predicted value (95% CI)	70% (54–82%)	38% (29–48%)	32% (24–40%)

**Table 3:** Multiple logistic regression analysis to predict 30-day mortality. CT = computer tomography; APACHE = Acute Physiology and Chronic Health Evaluation.

Variable	Odds Ratio	95% Confidence Interval	*p*
Serum CCCK-18 (µ/L)	1.02	1.01–1.03	<0.001
Sex (female vs. male)	5.77	1.17–28.43	0.03
CT classification (high vs. low risk of death)	3.61	0.99–13.22	0.052
APACHE-II score (points)	1.38	1.17–1.63	<0.001

## References

[1] Brain Trauma Foundation, American Association of Neurological Surgeons, Congress of Neurological Surgeons (2007). Guidelines for the management of severe traumatic brain injury. J. Neurotrauma.

[2] Cavallucci V., D’Amelio M. (2011). Matter of life and death: The pharmacological approaches targeting apoptosis in brain diseases. Curr. Pharm. Des..

[3] Wang K., Liu B., Ma J. (2014). Research progress in traumatic brain penumbra. Chin. Med. J. (Engl).

[4] Rovegno M., Soto P.A., Sáez J.C., von Bernhardi R. (2012). Biological mechanisms involved in the spread of traumatic brain damage. Med. Intensiva..

[5] Kunz A., Dirnagl U., Mergenthaler P. (2010). Acute pathophysiological processes after ischaemic and traumatic brain injury. Best Pract. Res. Clin. Anaesthesiol..

[6] Raghupathi R., Conti A.C., Graham D.I., Krajewski S., Reed J.C., Grady M.S., Trojanowski J.Q., McIntosh T.K. (2002). Mild traumatic brain injury induces apoptotic cell death in the cortex that is preceded by decreases in cellular Bcl-2 immunoreactivity. Neuroscience.

[7] Villapol S., Byrnes K.R., Symes A.J. (2014). Temporal dynamics of cerebral blood flow, cortical damage, apoptosis, astrocyte-vasculature interaction and astrogliosis in the pericontusional region after traumatic brain injury. Front. Neurol..

[8] Chen R., Wang J., Jiang B., Wan X., Liu H., Liu H., Yang X., Wu X., Zou Q., Yang W. (2013). Study of cell apoptosis in the hippocampus and thalamencephalon in a ventricular fluid impact model. Exp. Ther. Med..

[9] Clark R.S., Kochanek P.M., Chen M., Watkins S.C., Marion D.W., Chen J., Hamilton R.L., Loeffert J.E., Graham S.H. (1999). Increases in Bcl-2 and cleavage of caspase-1 and caspase-3 in human brain after head injury. FASEB J..

[10] Miñambres E., Ballesteros M.A., Mayorga M., Marin M.J., Muñoz P., Figols J., López-Hoyos M. (2008). Cerebral apoptosis in severe traumatic brain injury patients: An in vitro, in vivo, and postmortem study. J. Neurotrauma.

[11] Chu P.G., Weiss L.M. (2002). Keratin expression in human tissues and neoplasms. Histopathology.

[12] Caulín C., Salvesen G.S., Oshima R.G. (1997). Caspase cleavage of keratin 18 and reorganization of intermediate filaments during epithelial cell apoptosis. J. Cell Biol..

[13] Lorente L., Martín M.M., González-Rivero A.F., Ferreres J., Solé-Violán J., Labarta L., Díaz C., Jiménez A., Borreguero-León J.M. (2014). Serum levels of caspase-cleaved cytokeratin-18 and mortality are associated in severe septic patients: Pilot study. PLoS One.

[14] Lorente L., Rodriguez S.T., Sanz P., Pérez-Cejas A., Padilla J., Díaz D., González A., Martín M.M., Jiménez A., Barrera M.A. (2016). Prognostic value of serum caspase-cleaved cytokeratin-18 levels before liver transplantation for one-year survival of patients with hepatocellular carcinoma. Int. J. Mol. Sci..

[15] Luciani P., Gelmini S., Ferrante E., Lania A., Benvenuti S., Baglioni S., Mantovani G., Cellai I., Ammannati F., Spada A. (2005). Expression of the antiapoptotic gene seladin-1 and octreotide-induced apoptosis in growth hormone-secreting and nonfunctioning pituitary adenomas. J. Clin. Endocrinol. Metab..

[16] Ari F., Aztopal N., Oran S., Bozdemir S., Celikler S., Ozturk S., Ulukaya E. (2014). Parmelia sulcata Taylor and Usnea filipendula Stirt induce apoptosis-like cell death and DNA damage in cancer cells. Cell Prolif..

[17] Yuan Z.G., Wang J.L., Jin G.L., Yu X.B., Li J.Q., Qiu T.L., Dai R.X. (2015). Serum caspase-cleaved cytokeratin-18 levels and outcomes after aneurysmal subarachnoid hemorrhage. J. Neurol. Sci..

[18] Gu S.J., Lu M., Xuan H.F., Chen X.Z., Dong W.F., Yan X.F., Si Y., Gao G.L., Hu D.X., Miao J.Q. (2016). Predictive value of serum caspase-cleaved cytokeratin-18 concentrations after acute intracerebral hemorrhage. Clin. Chim. Acta.

[19] Lorente L., Martín M.M., Pérez-Cejas A., Ramos L., Argueso M., Solé-Violán J., Cáceres J.J., Jiménez A., García-Marín V. (2018). Association between serum levels of caspase-cleaved cytokeratin-18 and early mortality in patients with severe spontaneous intracerebral hemorrhage. BMC Neurosci..

[20] Lorente L., Martín M.M., Pérez-Cejas A., Ramos L., Argueso M., Solé-Violán J., Cáceres J.J., Jiménez A., García-Marín V. (2018). High serum levels of caspase-cleaved cytokeratin-18 are associated with malignant middle cerebral artery infarction patient mortality. BMC Neurol..

[21] Lorente L., Martín M.M., González-Rivero A.F., Argueso M., Ramos L., Solé-Violán J., Cáceres J.J., Jiménez A., Borreguero-León J.M. (2015). Serum levels of caspase-cleaved cytokeratin-18 in patients with severe traumatic brain injury are associated with mortality: A pilot study. PLoS One.

[22] Teasdale G., Jennett B. (1974). Assessement of coma and impaired conciousness. A practical scale. Lancet.

[23] Baker S.P., O’Neill B., Haddon W., Jr Long W.B. (1974). The injury severity score: A method for describing patients with multiple injuries and evaluating emergency care. J. Trauma.

[24] Knaus W.A., Draper E.A., Wagner D.P., Zimmerman J.E. (1985). APACHE II: A severity of disease classification system. Crit. Care. Med..

[25] Marshall L.F., Marshall S.B., Klauber M.R., Van Berkum Clark M., Eisenberg H., Jane J.A., Luerssen T.G., Marmarou A., Foulkes M.A. (1992). The diagnosis of head injury requires a classification based on computed axial tomography. J. Neurotrauma.

[26] Lu J., Goh S.J., Tng P.Y., Deng Y.Y., Ling E.A., Moochhala S. (2009). Systemic inflammatory response following acute traumatic brain injury. Front. Biosci. (Landmark Ed.).

[27] Wesche-Soldato D.E., Swan R.Z., Chung C.S., Ayala A. (2007). The apoptotic pathway as a therapeutic target in sepsis. Curr. Drug Targets.

[28] Saykally J.N., Rachmany L., Hatic H., Shaer A., Rubovitch V., Pick C.G., Citron B.A. (2012). The nuclear factor erythroid 2-like 2 activator, *tert*-butylhydroquinone, improves cognitive performance in mice after mild traumatic brain injury. Neuroscience.

[29] Abrahamson E.E., Ikonomovic M.D., Ciallella J.R., Hope C.E., Paljug W.R., Isanski B.A., Flood D.G., Clark R.S., DeKosky S.T. (2006). Caspase inhibition therapy abolishes brain trauma-induced increases in Abeta peptide: Implications for clinical outcome. Exp. Neurol..

[30] Soustiel J.F., Palzur E., Nevo O., Thaler I., Vlodavsky E. (2005). Neuroprotective anti-apoptosis effect of estrogens in traumatic brain injury. J. Neurotrauma.

[31] Clausen F., Lundqvist H., Ekmark S., Lewén A., Ebendal T., Hillered L. (2004). Oxygen free radical-dependent activation of extracellular signal-regulated kinase mediates apoptosis-like cell death after traumatic brain injury. J. Neurotrauma.

[32] Clark R.S., Kochanek P.M., Watkins S.C., Chen M., Dixon C.E., Seidberg N.A., Melick J., Loeffert J.E., Nathaniel P.D., Jin K.L. (2000). Caspase-3 mediated neuronal death after traumatic brain injury in rats. J. Neurochem..

[33] Sanchez Mejia R.O., Ona V.O., Li M., Friedlander R.M. (2001). Minocycline reduces traumatic brain injury-mediated caspase-1 activation, tissue damage, and neurological dysfunction. Neurosurgery.

[34] Xue Z., Song Z., Wan Y., Wang K., Mo L., Wang Y. (2017). Calcium-sensing receptor antagonist NPS2390 attenuates neuronal apoptosis though intrinsic pathway following traumatic brain injury in rats. Biochem. Biophys. Res. Commun..

[35] Yang H., Gu Z.T., Li L., Maegele M., Zhou B.Y., Li F., Zhao M., Zhao K.S. (2017). SIRT1 plays a neuroprotective role in traumatic brain injury in rats via inhibiting the p38 MAPK pathway. Acta Pharmacol. Sin..

[36] Hartings J.A., Bullock M.R., Okonkwo D.O., Murray L.S., Murray G.D., Fabricius M., Maas A.I., Woitzik J., Sakowitz O., Mathern B. (2011). Spreading depolarisations and outcome after traumatic brain injury: A prospective observational study. Lancet Neurol..

[37] Fabricius M., Fuhr S., Bhatia R., Boutelle M., Hashemi P., Strong A.J., Lauritzen M. (2006). Cortical spreading depression and peri-infarct depolarization in acutely injured human cerebral cortex. Brain.

[38] Jahanbazi Jahan-Abad A., Alizadeh L., Sahab Negah S., Barati P., Khaleghi Ghadiri M., Meuth S.G., Kovac S., Gorji A. (2018). Apoptosis Following Cortical Spreading Depression in Juvenile Rats. Mol. Neurobiol..

